# Analysis of BeiDou Satellite Measurements with Code Multipath and Geometry-Free Ionosphere-Free Combinations

**DOI:** 10.3390/s16010123

**Published:** 2016-01-20

**Authors:** Qile Zhao, Guangxing Wang, Zhizhao Liu, Zhigang Hu, Zhiqiang Dai, Jingnan Liu

**Affiliations:** 1 GNSS Research Center, Wuhan University, No.129 Luoyu Road, Wuhan 430079, China; zhaoql@whu.edu.cn (Q.Z.); zhigang.hu@whu.edu.cn (Z.H.); jnliu@whu.edu.cn (J.L.); 2 Department of Land Surveying & Geo-Informatics (LSGI), The Hong Kong Polytechnic University (PolyU), 181 Chatham Road South, Hung Hom, Kowloon, Hong Kong, China; 3 School of Geodesy and Geomatics, Wuhan University, No.129 Luoyu Road, Wuhan 430079, China; dzq@whu.edu.cn

**Keywords:** BDS, GNSS, CNR, multipath, GFIF, elevation, hardware delay

## Abstract

Using GNSS observable from some stations in the Asia-Pacific area, the carrier-to-noise ratio (CNR) and multipath combinations of BeiDou Navigation Satellite System (BDS), as well as their variations with time and/or elevation were investigated and compared with those of GPS and Galileo. Provided the same elevation, the CNR of B1 observables is the lowest among the three BDS frequencies, while B3 is the highest. The code multipath combinations of BDS inclined geosynchronous orbit (IGSO) and medium Earth orbit (MEO) satellites are remarkably correlated with elevation, and the systematic “V” shape trends could be eliminated through between-station-differencing or modeling correction. Daily periodicity was found in the geometry-free ionosphere-free (GFIF) combinations of both BDS geostationary Earth orbit (GEO) and IGSO satellites. The variation range of carrier phase GFIF combinations of GEO satellites is −2.0 to 2.0 cm. The periodicity of carrier phase GFIF combination could be significantly mitigated through between-station differencing. Carrier phase GFIF combinations of BDS GEO and IGSO satellites might also contain delays related to satellites. Cross-correlation suggests that the GFIF combinations’ time series of some GEO satellites might vary according to their relative geometries with the sun.

## 1. Introduction

The BeiDou Navigation Satellite System (BDS, earlier referred to as COMPASS) has been providing position, navigation, and timing (PNT) services since 27 December 2012, covering the whole Asia-Pacific region. The system currently consists of 15 satellites, including five geostationary Earth orbit (GEO) satellites, five inclined geosynchronous orbit (IGSO) satellites, and five medium Earth orbit (MEO) satellites. It will eventually provide PNT services for worldwide users by the end of 2020, comprising five GEO, three IGSO, and twenty-seven MEO satellites [1].

Like the other Global Navigation Satellite Systems (GNSS), the BDS has a significant potential in many applications and scientific research community. To make a better use of the BDS system, the BDS performance and signal characteristics must be fully evaluated and understood. In earlier studies, it has been shown that Precise Point Positioning (PPP) result using BDS observations alone is comparable with that using Global Positioning System (GPS) only, and that PPP convergence speed and positioning accuracy could be improved after integrating BDS with GPS observations [2,3]. With triple-frequency observables available, characteristics of combined measurements [4] and ambiguity resolution with triple-frequency carrier phase [5,6,7] were also investigated. It has been commonly agreed that severe multipath effects slow down the convergence of PPP and degrade the reliability of PPP ambiguity resolution [5,6,8,9]. Methods for mitigating multipath effects on both code [10,11] and carrier phase measurements [12] have been developed.

Previous studies showed that BDS observables of higher elevations have higher carrier-to-noise ratio (CNR) and smaller variation in multipath combinations [4,10,11,13]. With the combination of GPS and BDS, high cut-off elevation angles, *i.e.*, up to 30° for the single-frequency cases or 40° for the dual-frequency, could still guarantee high success-rate (nearly 100%) of ambiguity resolution in RTK positioning [14]. In addition, it was shown that multipath combinations of BDS observables display systematic variations with elevations, which is significantly different from that of GPS, GLONASS, or Galileo [10,11]. This multipath variation should be taken into account since it affects the convergence and reliability of ambiguity resolution of BDS data.

Our analysis finds that multipath combination of BDS observables contains not only real multipath effects but also some other multipath-like biases. Understanding and proper handling of these biases and their characteristics, as well as their difference from GPS, will help correct BDS signal errors and improve the positioning accuracy of using BDS or multi-GNSS data.

This paper is organized as below. The mathematical models for GNSS code multipath combination and geometry-free ionosphere-free (GFIF) combination are given in Section 2. In Section 3, the BDS satellite CNR and multipath combination are investigated and compared with those of GPS and Galileo satellites. Cross-correlation analyses are conducted between multipath of different frequencies, between multipath and elevation, as well as between multipath and azimuth. To further examine the elevation-dependent bias in the multipath of BDS IGSO and MEO satellites, single-differencing between two stations is conducted. The GFIF combination of BDS satellites is also investigated. The conclusions are given in Section 4.

## 2. Mathematical Models

Multipath effects are due to the reflections of satellite signals from nearby objects, such as trees, walls, and water surfaces. Although multipath effects are usually considered to occur at the receiver end, it is worth noting that the “nearby objects” here can also refer to the possible reflectors at the satellite transmitters, such as the solar panels or so. The multipath effects on code measurements could be investigated through the so-called multipath combination [15,16,17], which will be discussed below. To assess the errors of carrier phases, the raw observations of carrier phases on three different frequencies are usually employed to form the GFIF combination [13].

### 2.1. BDS Code Multipath Estimation

GNSS code multipath effects can be estimated through a combination of code range and carrier phase GNSS observables. Provided that dual- or triple-frequency observations are available, multipath combination related to code range on frequency i can be expressed as [11,15,16,17,18]:

(1) $$\kappa_{i}=p_{i}-\frac{f_{i}^{2}+f_{j}^{2}}{f_{i}^{2}-f_{j}^{2}}\lambda_{i}\varphi_{i}+\frac{2f_{j}^{2}}{f_{i}^{2}-f_{j}^{2}}\lambda_{j}\varphi_{j}$$

where p (in unit of meter) and φ (in unit of cycle) represent the code range and carrier phase observables, respectively; fand λ are the frequency and wavelength, respectively; and the subscripts i, j (i≠j) are used to denote different frequencies. $\kappa_{i}$ contains not only multipath effect on $p_{i}$, but also the terms of ambiguities and hardware delays. Assuming no cycle slip has occurred or the cycle slip has been corrected [19], if any, a zero-mean term for multipath effect on frequency i could be derived as [11]:

(2) $$MP_{i}=\kappa_{i}-{\bar{\kappa }}_{i}$$

where ${\bar{\kappa }}_{i}$ is the average of $\kappa_{i}$ over time. ${\bar{\kappa }}_{i}$ may also absorb constant component of multipath effects, if any. For simplicity, subscripts for receiver, satellite, and epoch time have been omitted in Equation (2).

The theoretical maximum multipath effect on carrier phase observable is only one quarter of carrier wave cycle [11,15,20]. For BDS satellites, the maximum multipath effects for BDS B1, B2, and B3 carrier phase measurements are 4.8 cm, 6.2 cm and 5.9 cm, respectively. Compared with the size of code multipath that is at the level of several meters, the multipath effects on carrier phase are negligible [11].

### 2.2. GFIF Combination for Carrier Phase Bias Analysis

For a receiver-satellite pair, the ionosphere-free (IF) linear combination of dual-frequency code and carrier phase observations can be written as [15,21]:

(3) $$\mathrm{IF}(\varphi_{i},\varphi_{j})=\frac{f_{i}^{2}}{f_{i}^{2}-f_{j}^{2}}\varphi_{i}-\frac{f_{j}^{2}}{f_{i}^{2}-f_{j}^{2}}\varphi_{j}=\rho +\lambda_{c}N_{c}+b_{\varphi }-B_{\varphi }$$

where ρ is the sum of all time-dependent and frequency-independent (or non-dispersive) items, mainly including the receiver-satellite range, receiver clock error, satellite clock error, and tropospheric error; $N_{c}$ and $\lambda_{c}$ are the ambiguity and corresponding wavelength of IF combination; $b_{\varphi }$ and $B_{\varphi }$ are the receiver and satellite hardware delays in carrier phase measurements, respectively.

The BDS satellites broadcast signals at B1, B2, and B3 three frequencies of 1561.098 MHz, 1207.140 MHz, and 1268.520 MHz, respectively. Given the triple-frequency observables in BDS, a geometry-free and ionosphere-free (GFIF) combination can be formed [13]:

(4) $$\mathrm{GFIF}(\varphi_{1},\varphi_{2},\varphi_{3})=\mathrm{IF}(\varphi_{1},\varphi_{2})-\mathrm{IF}(\varphi_{1},\varphi_{3})=-0.457\cdot \varphi_{1}-1.487\cdot \varphi_{2}+1.944\cdot \varphi_{3}$$

The coefficients −0.457, −1.487, and 1.944 correspond to the BDS frequencies B1, B2, and B3, respectively. The GFIF combination mainly includes a weighted sum of receiver noise, multipath, and hardware delay in each frequency and a combination of carrier phase ambiguities. The high-order ionosphere, phase wind-up, and phase center variations in the GFIF combination is sufficiently small and they can be neglected [13,21]. Equation (4) is formed only with carrier phase observables, thus both noise level and multipath effect are much lower. It is, thus, suitable for investigating the characteristics of hardware delays in BDS carrier phase observables.

## 3. Experimental Analysis

**Figure 1:**
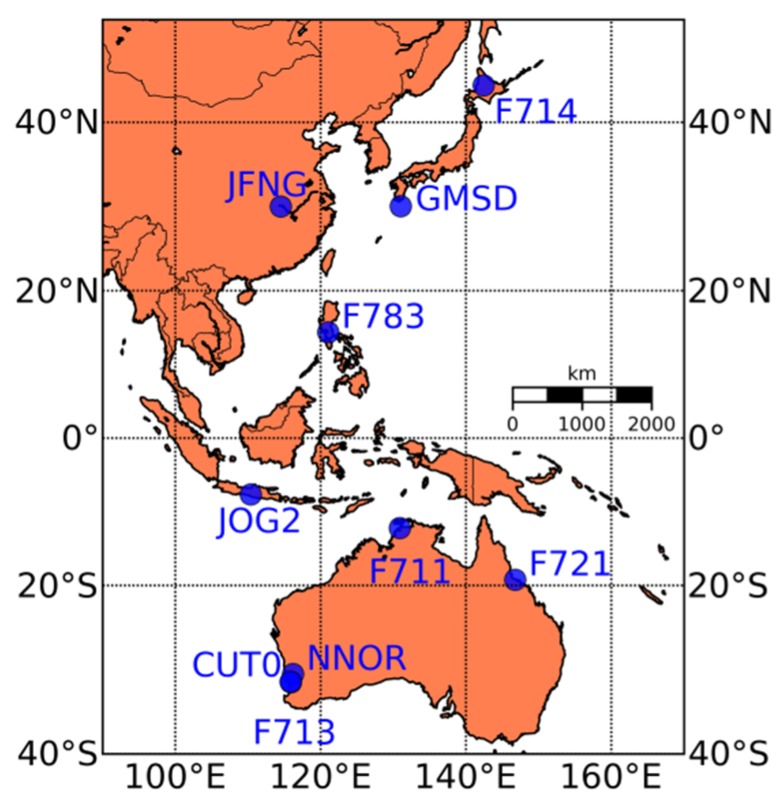
The distribution of the GNSS stations.

In the analysis, multi-GNSS data collected from 19 to 28 May 2013 at 10 stations in the Asia-Pacific region were employed. The sampling interval was set as 30 s. The geographic distribution of these GNSS stations is shown in Figure 1. Listed in Table 1 are the antenna types and receiver types of the 10 stations, eight of which are equipped with TRIMBLE NETR9 receivers. Unfortunately, primary investigation shows that no triple-frequency BDS observable is available at the stations JOG2 and NNOR, so the following analyses, especially those relevant to BDS measurements, is mainly based on the data from TRIMBLE NET9 receivers.

**Table 1:** The types of antennas and receivers equipped at the GNSS stations.

Stations	Antenna Types	Receiver Types
**CUT0**	TRM59800.00 SCIS	TRIMBLE NETR9 4.70
**F711**	TRM57971.00 NONE	TRIMBLE NETR9
**F713**	TRM55971.00 NONE	TRIMBLE NETR9
**F714**	TRM55971.00 NONE	TRIMBLE NETR9
**F721**	TRM57971.00 NONE	TRIMBLE NETR9
**F783**	TRM57971.00 NONE	TRIMBLE NETR9
**GMSD**	TRM59800.00 SCIS	TRIMBLE NETR9 4.80
**JFNG**	TRM59800.00 NONE	TRIMBLE NETR9 4.70
**JOG2**	JAV_RINGANT_G3T NONE	JAVAD TRE_G3TH DELTA3.4.7
**NNOR**	SEPCHOKE_MC NONE	SEPT POLARX4 2.3.4

### 3.1. Comparing CNR and Code Multipath Combinations of Different Systems

GPS, GLONASS, Galileo, and BDS are considered the main four global navigation satellite systems at present. Although they share the principle of distance resection to provide service, they are different in some aspects, such as the constellation, frequency choice, multi-access techniques, and so on. The space segment of BDS comprises GEO, IGSO, and MEO satellites. Unlikely, the constellations of the other three systems contain MEO satellites only. The technology of frequency division multi-access (FDMA) is employed by GLONASS, while that of code division multi-access (CDMA) by the other three. In addition, the frequencies of signals are different from system to system, and triple-frequency observables are already available for BDS and Galileo systems. Considering the above differences, behaviors of carrier-to-noise ratios and code multipath combinations for the four different systems are compared.

**Figure 2:**
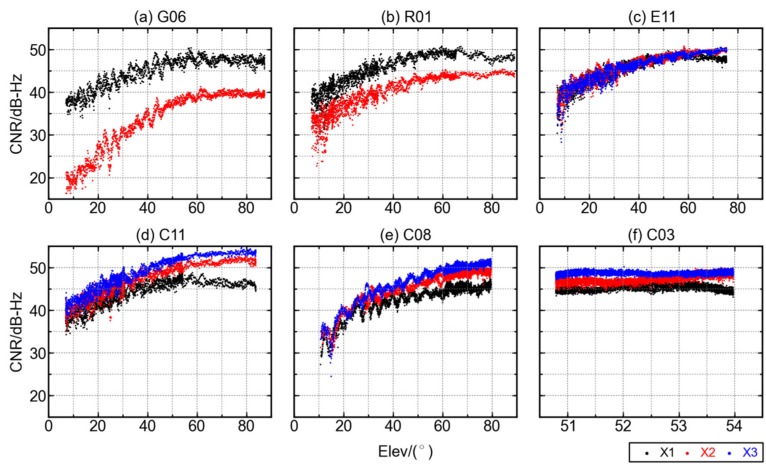
The relationship between CNRs and satellite elevations for GPS, GLONASS, Galileo, and BDS satellites at GNSS station F713. (**a**) G06; (**b**) R01; (**c**) E11; (**d**) C11; (**e**) C08; (**f**) C03. Three frequencies are denoted as X1, X2, and X3 with the colors black, red and blue.

Carrier-to-noise ratio (CNR) can indicate the precision of carrier phase observations so it is utilized to evaluate the quality of ranging signals. Taking the station F713 for instance, observables of GPS, GLONASS, Galileo, and BDS were simultaneously collected during the same period. The CNRs for observables of GPS satellite (G06), GLONASS satellite (R01), Galileo satellite (E11), and three BDS satellites (C03, C08, and C11) are illustrated in Figure 2 for the station F713. BDS C03 and C08 are GEO and IGSO satellites, respectively, while the C11, as well as GPS and Galileo satellites, are all MEO ones. Different colors represent different frequencies of each system.

As is shown in Figure 2, the maximum CNRs for all GNSS satellites are around 50 dB-Hz, and the CNRs vary significantly with the elevations for all satellites except the BDS GEO ones, due to their relatively stable elevations. For GPS satellite G06 and GLONASS satellite R01, the CNR of measurement on the first frequency is higher than that on the second, provided the same elevation. However, it is not always true for all GLONASS satellites, some of which, not displayed in the figure, have almost the same CNR for measurement on both frequencies. For BDS satellites, the B1 measurement has the lowest CNR among all the three frequencies while the B3 has the highest CNR. The CNR of B3 measurement of satellite C11 reaches almost 55 dB-Hz at high elevation angles (>60°). No apparent difference is found in the CNRs of triple-frequency observables of Galileo satellite. When the elevation of satellite is relatively low, the CNR of GPS L2 observables is more sensitive to the elevation than all the other three systems. The CNR of GPS L2 observable is only about 20 dB-Hz at elevation below 10°.

Figure 2 shows that the CNRs of all satellites, except C03, increase significantly with the increase of elevations. This is consistent with previous studies about GNSS measurement CNR and the elevation of corresponding satellite [10,11,13]. The stable CNR of satellite C03 is due to its unique GEO satellite orbit, which is designed to appear stationary above the equator. Though the orbit of GEO satellites is affected by perturbations such as solar radiation, the actual geometry variation between the GEO satellites and the earth is still small. As an example, the elevation of GEO satellite C03 observed at station F713 has a variation of only 3°.

To compare the code multipath effects among different GNSS systems, the multipath combinations were estimated from GPS, GLONASS, Galileo, and BDS code observations with Equation (2). To make the comparison fair, only the MEO satellites were selected to represent BDS, since the satellites of all the other three systems are MEO ones. The behaviors of multipath combinations for GEO and IGSO satellites of BDS will be discussed in the following sections.

**Figure 3:**
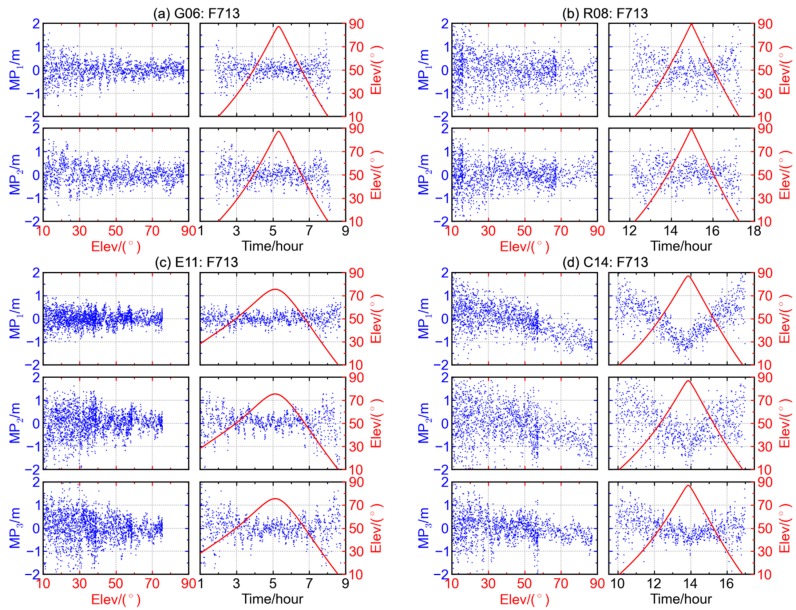
The multipath combinations (blue) and satellite elevations (red) for (**a**) GPS satellite G06, (**b**) GLONASS satellite R08, (**c**) Galileo satellite E11 and (**d**) BDS MEO satellite C14 at station F713.

The code multipath combinations of G06, R08, E11, and C14 are shown as Figure 3a–d, respectively. Each satellite represents a GNSS system, and the different rows of each figure represent the multipath combinations for different frequencies. The left panels of each figure show the variations of multipath combinations with respect to elevation, while the right panels show the time series of multipath combination (blue) and elevation angle (red). It can be seen that both the amplitude and dispersion of code multipath combination decrease significantly with the increase of satellite elevation. When the elevation is low, the amplitude of code multipath combination is about 2.0 m and it drops to below 1.0 m when the elevation is over 60°. The code multipath on the first frequency of Galileo satellite E11 has the smallest amplitude and dispersion, for which the multipath magnitude is below 1.0 m, even at low elevation angles.

The average of code multipath combination varies from about 0.5 m at low elevation to about −1.0 m at high elevation. Compared with Figure 3a–c, a deep trough can be clearly seen in each multipath time series when the satellite has the maximum elevation in the right three panels of Figure 3d. Correspondingly, the left three panels of Figure 3d show that the numerical values of code multipath combination of BDS MEO satellite C14 decrease when the elevation increases, suggesting the existence of elevation-dependent biases. The similar case also holds for the other BDS MEO satellites whose results are not displayed here, and the elevation-dependent trends of B1 and B2 code multipath combinations are usually more significant than that of B3.

### 3.2. Bias in Code Multipath Combination of BDS

**Figure 4:**
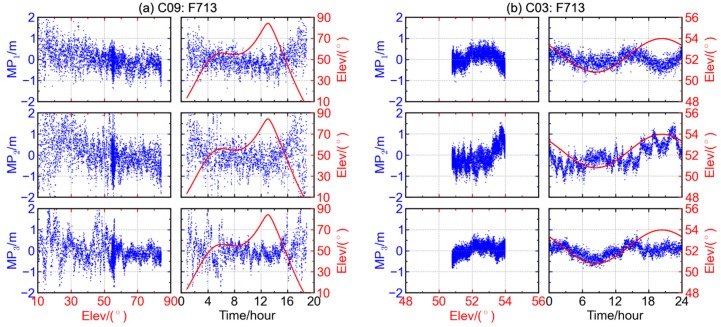
The relationship between multipath combination (blue) and satellite elevations (red) for BDS (**a**) IGSO satellite C09 and (**b**) GEO satellite C03 at station F713.

The above discussion shows that the code multipath combinations of BDS MEO satellite contain remarkable elevation-dependent bias, which is not seen in those of GPS, GLONASS, or Galileo satellites. Considering the difference in orbit types of BDS satellites, we also examine the elevation-dependent bias phenomenon with the BDS IGSO and GEO satellites. Similar phenomenon is also observed in the code multipath combinations of BDS IGSO satellites (see Figure 4a), though the trends for IGSO satellites are not as significant as the MEO ones due to relatively slow variation of IGSO satellite elevation.

When it comes to BDS GEO satellites (see Figure 4b), the multipath time series show daily periodicity instead of elevation-dependent bias. This is consistent with the results of Fast Fourier transformation and time correlation analysis which indicate that the principal period for code multipath time series of BDS GEO satellites is roughly equal to a sidereal day [11]. The diurnal periodicity could be exploited to mitigate the code multipath effects of BDS GEO satellites. For example, the low-frequency components of code multipath combinations in history could be extracted and employed as an empirical model to correct the observables. It is proved that the precision of single point positioning could be improved after applying such corrections [11].

**Figure 5:**
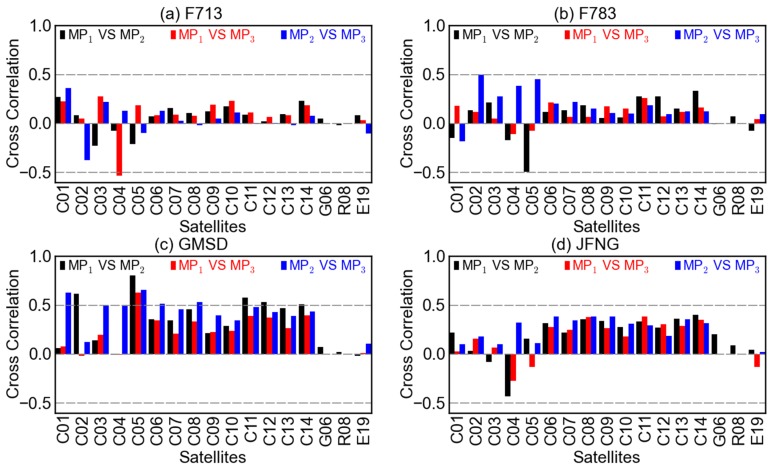
Cross-correlations between the code multipath combinations $MP_{1}$ and $MP_{2}$ (black), $MP_{1}$and $MP_{3}$ (red), $MP_{2}$ and $MP_{3}$ (blue) at stations (**a**) F713, (**b**) F783, (**c**) GMSD, and (**d**) JFNG.

**Figure 6:**
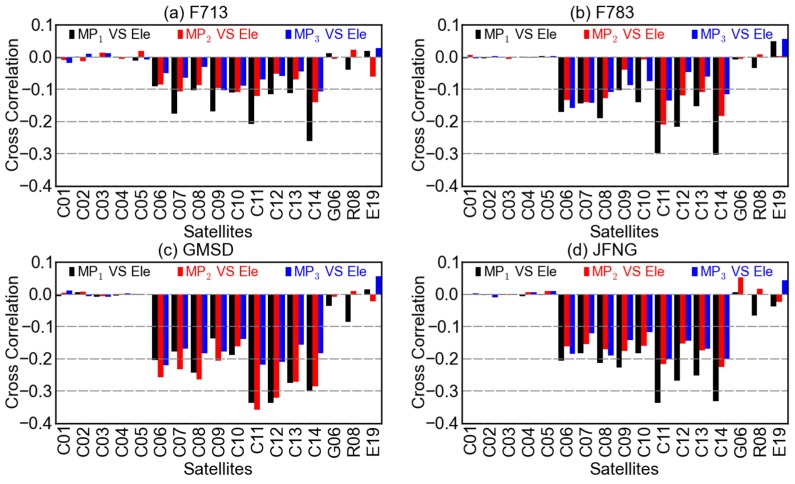
Cross-correlations between the code multipath combinations $MP_{1}$ (black), $MP_{2}$ (red), $MP_{3}$ (blue) and the elevations at stations (**a**) F713, (**b**) F783, (**c**) GMSD, and (**d**) JFNG.

**Figure 7:**
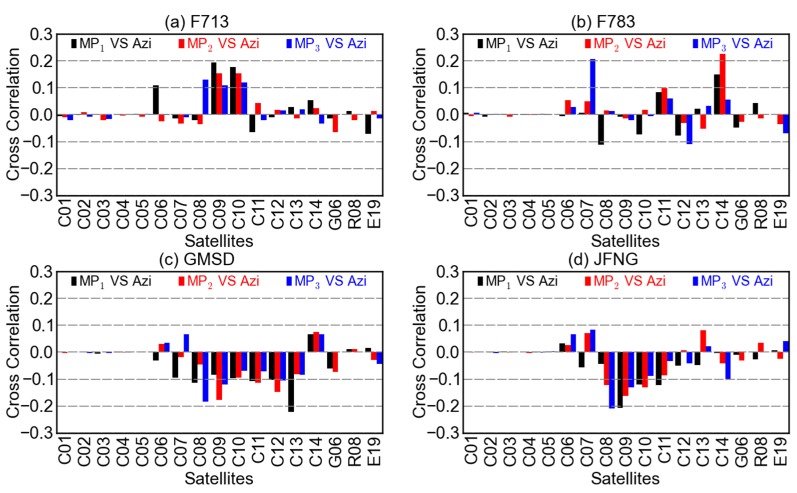
Cross-correlations between the code multipath combinations $MP_{1}$ (black), $MP_{2}$ (red), $MP_{3}$ (blue) and the azimuths at stations (**a**) F713, (**b**) F783, (**c**) GMSD, and (**d**) JFNG.

As is shown in Figure 3d and Figure 4, the code multipath combination time series on different frequencies of BDS MEO and IGSO satellites show almost the same pattern, while the patterns of the counterparts of GEO satellite are conspicuously different from frequency to frequency. To know more about the between-frequency relationships of multipath, correlations analyses were performed between multipath combinations of every two frequencies. Shown in Figure 5 are the cross-correlations at the four stations F713, F783, GMSD, and JFNG. In addition to the cross-correlation results of all available BDS satellites, those of GPS satellite G06 and Galileo satellite E19 are also displayed for comparison. For GPS satellites, only $MP_{1}$ and $MP_{2}$ are available, and the similar case also holds for the following Figure 6 and Figure 7.

In Figure 5, the between-frequency correlations of BDS GEO satellites (*i.e.*, C01–C05) can have high absolute values, but they seem random considering the differences among satellites and stations. When it comes to the cases of BDS IGSO (*i.e.*, C06–C10) and MEO (*i.e.*, C11–C14) satellites,the correlations behave somewhat systematically. In each panel, the directions of the bars for IGSO and MEO satellites are almost the same, especially for the cross-correlations of $MP_{1}$ *vs.* $MP_{2}$ and $MP_{1}$ *vs.* $MP_{3}$. When comparing among different GNSS systems, it can be seen that the between-frequency correlations for GPS and Galileo satellites are relatively low and random. When comparing among different stations, it is noted that the between-frequency correlations at stations GMSD and JFNG are generally higher than those at stations F713 and F783. This is probably due to the different antenna types.

Show in Figure 6 are cross-correlations between code multipath combinations and the elevations at different stations. For BDS GEO satellites, the correlations are quite low and their directions are random considering different frequencies, satellites, or stations. So, too, is it with GPS and Galileo satellites. For BDS IGSO and MEO satellites, however, the code multipath and elevation are more or less negatively correlated, although the correlation coefficients are quantitatively different for different satellites or stations. These results, to some extent, validate the existence of elevation-dependent biases in code multipath combinations of BDS non-GEO satellites. It is also suggested that the multipath effects of the GEO and non-GEO satellites might need discriminative treatments, taking their remarkably different behaviors into account. For example, the method of elevation-dependent model might be feasible but only for the non-GEO satellites.

Unlikely, the correlations between code multipath combinations of non-GEO satellites and their azimuths are much more random, as is shown in Figure 7. Since the relationship of multipath and azimuth is more likely to be affected by the station surroundings, the cross-correlation of multipath and azimuth of a certain satellite could be of great difference from station to station. For BDS GEO satellites, the cross-correlations in Figure 7 are similar to those in Figure 6, both small and random, which could be explained by the relatively stable geometry between the GEO satellites and stations.

**Figure 8:**
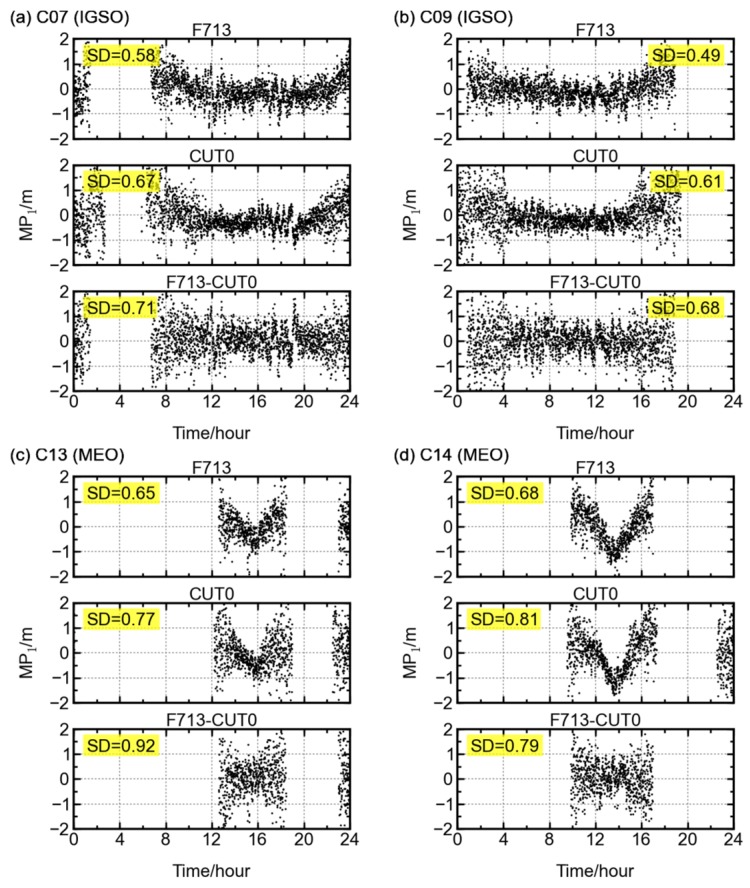
The systematic bias in multipath combination is eliminated through a between-station differencing. The single-station elevation-angle dependent troughs for satellites (**a**) C07, (**b**) C09, (**c**) C13, and (**d**) C14 vanish after the between-station differencing is performed.

To further examine the elevation-dependent biases in the BDS IGSO and MEO satellite signals, the code multipath time series of each satellite at stations F713 (31.94° S, 115.84° E) and CUT0 (32.00° S, 115.89° E) are compared and between-station differencing is conducted. Shown in Figure 8 are the time series of single-station and between-station differencing multipath combinations obtained from the B1 frequency of IGSO satellites C07 and C09 and MEO satellites C13 and C14. The code multipath time series at the two stations show similar elevation-dependent “V” shape patterns. The troughs in the multipath time series vanish after the between-station differencing is performed. For the other two BDS frequencies B2 and B3, and for the other BDS IGSO and MEO satellites, similar results can be obtained in their multipath time series. Their “V” shape in multipath time series can be significantly reduced by performing differencing between two stations.

The distance between the F713 and CUT0 stations is about 10 km, and the surroundings of two stations are distinctly different [11]. Thus, the above results partly confirm that the elevation-dependent bias is related to satellite instead of station. In this sense, the code multipath combination of BDS satellites might not only contain multipath effects from the surroundings of the stations, but also contain some multipath-like biases from the satellites. One of the possible explanations could be that the BDS signals subject to multipath effects caused by reflections of the satellite components, such as the solar panels. In addition, the internal hardware delay could also be the contributor to the multipath-like biases. However, no data are available to verify this at the moment.

**Figure 9:**
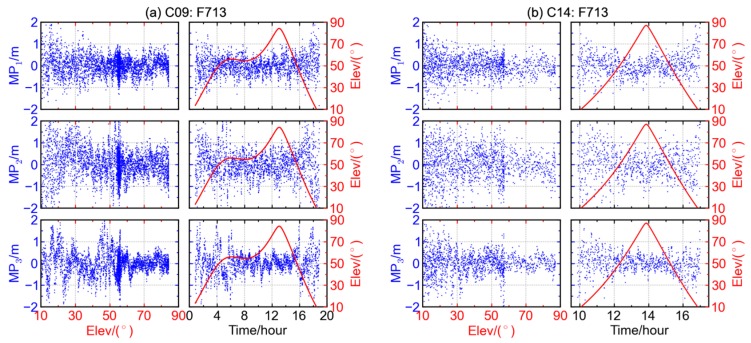
The multipath combinations of BDS satellites (**a**) C09 (IGSO) and (**b**) C14 (MEO); after the elevation-dependent correction model is applied.

Although high cut-off elevation angles could, to some extent, help circumvent the effects from multipath, it will lead to the loss of the availability [14]. An alternative method of dealing with the biases in multipath combination is to correct it with a proper model, considering the abovementioned high correlation between multipath combinations and the elevation of BDS non-GEO satellites. Wanninger and Beer (2014) introduced an elevation-dependent correction model for BDS IGSO and MEO satellites. Shown in Figure 9 are the multipath combinations of BDS satellites C09 (IGSO) and C14 (MEO) after applying the elevation-dependent correction model. The similar results can be obtained for the other IGSO and MEO satellites at other stations. With the correction, the elevation-dependent bias in multipath combination disappears. It reminds us that the precision of positioning could be improved by applying a proper elevation-dependent correction model to the raw observables of BDS IGSO and MEO satellites. Fortunately, the relationship of elevation and the bias for each non-GEO satellite is relatively stable, so an elevation-dependent correction model is expected to be also applied for kinematic or real-time cases. Therefore, efforts should be made to work out a delicate elevation-dependent correction model for each non-GEO satellite.

### 3.3. Biases in Carrier Phase Geometry-Free and Ionosphere-Free Combination

As discussed in Section 2, the carrier phase GFIF combination removes the frequency-independent terms and also the first-order ionospheric error. Only the ambiguity, measurement noises, and hardware delays remain in the combination. In order to examine the variation of observation noises, the ambiguity term is removed through averaging the GFIF combination observations.

**Figure 10:**
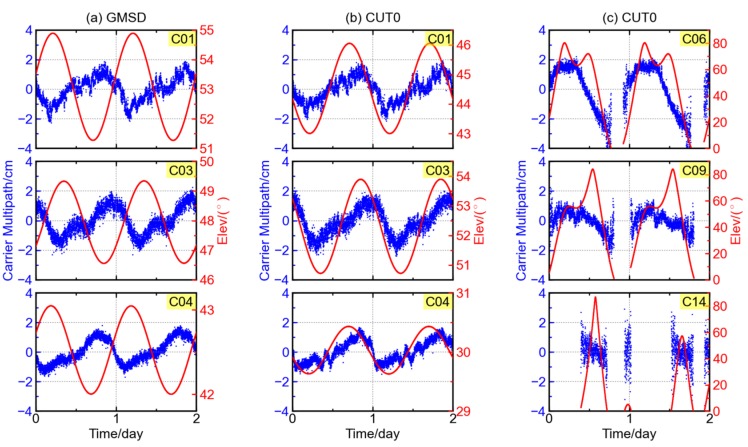
GFIF combination (blue) and elevation (red) of BDS satellites at stations (**a**) GMSD and (**b,c**) CUT0.

Figure 10 shows the GFIF carrier phase combinations of BDS GEO satellites C01, C03, and C04 at the stations GMSD and CUT0, as well as those of C06, C09, and C14 at CUT0. The two stations GMSD (30.56° N, 131.02° E) and CUT0 (32.00° S, 115.89° E) are located in the northern and southern hemispheres, respectively. Shown in each panel is the time series of GFIF (black) and elevation (red) over a two-day period.

It is interesting to note that the GFIF combination variation with satellite elevation at the GMSD station has a trend opposite to that at the CUT0 stations. In Figure 10a, the GFIF combination at GMSD station decreases with respect to elevation but it is an increase trend at CUT0 station in Figure 10b. To verify whether the opposite trends of GFIF variation are hemisphere-dependent, GFIF combinations of more stations are examined. In the northern hemisphere, the stations GMSD, JFNG, and F783 have similar GFIF variation trend, decreasing with elevation. The only exception is the C01 at station F714. In the southern hemisphere, the GFIF variation trend at stations CUT0, F711, and F721 are similar to each other too, increasing with elevation, while that of C01 at station F713 is exceptional. The GFIF carrier phase combination series of JOG2 or NNOR station is not examined as no triple-frequency BDS observables are available at either station. In addition, the GFIF time series of BDS IGSO satellites show significant diurnal periodicity, but no obvious trend with respect to elevation (see satellites C06 and C09 in Figure 10c); neither do those of BDS MEO satellites (see satellite C14 in Figure 10c).

The above results indicate that the variation of GFIF combination is not elevation-dependent. The plausible variation trends with respect to elevation in Figure 10a,b substantially reflect the diurnal periodicity in GFIF, since the elevation of each GEO satellite shows clear diurnal periodicity.

**Figure 11:**
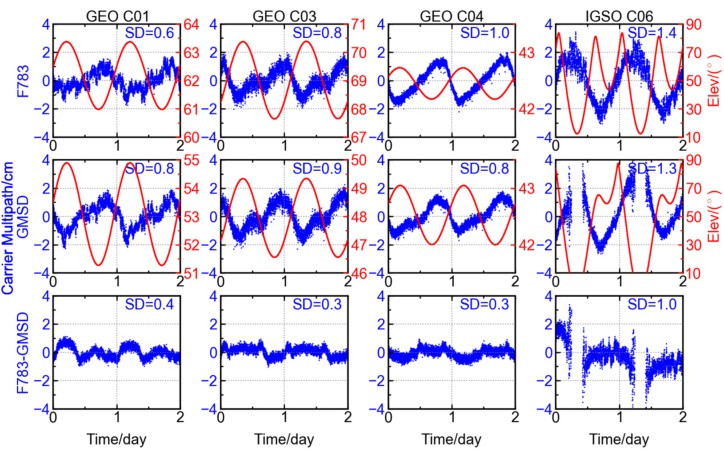
GFIF combinations at station F783 (**top** **row**), station GMSD (**middle** **row**) and differencing result between the two stations (**bottom** **row**) for different BDS satellites, the red curve showing the satellite elevation.

Similarly, the GFIF combinations at stations F783 (14.52° N, 121.02° E) and GMSD (30.56° N, 131.02° E) are selected to conduct the between-station-differencing. Here, the station F783 is employed instead of CUT0, since the stations F783 and GMSD, both located at the northern hemisphere, have longer time of common view for the BDS IGSO satellites. As shown in Figure 11, the variation amplitude of GFIF combinations for GEO and IGSO satellites are reduced from ~2.0 cm to ~1.0 cm after the differencing, and the standard deviations also decrease significantly, from ~1.0 cm to <0.4 cm. The same holds for IGSO satellite C06, whose standard deviation decreases from 1.4 cm to 1.0 cm after the single differencing between two stations.

The results in Figure 11 show that the trends contained in the GFIF combination of GEO and IGSO satellites can be, to a great extent, reduced through the single differencing between two stations. It should be noted that the two GNSS stations are distantly separated and their observation conditions are different and independent of each other. The common trends appearing in the GFIF combinations of the two stations should be attributed to the commonly observed satellite, unlikely from the stations. After the single differencing between two stations, there are still some variations of about 1.0 cm remaining in the GFIF time series. This could be explained by the high-order ionosphere and residuals of receiver-end hardware delays.

After an examination of Figure 10a,b, it is found that the phase shifts for the periodicities for the three GEO satellites are station-independent. Although the pattern of GFIF combination for each satellite may be different from station to station, the sequence in which the GFIF combinations get their crests or troughs is shared by different stations, *i.e.*, C04, C01, and C03. This order is consistent with how they are positioned above the equator from east to west. The longitudes for the GEO satellites C04, C01, and C03 are 160.0° E, 140.0° E, and 110.5° E [1,22,23]. Each satellite reaches the trough of its GFIF combination at around 1:00 P.M. in local time. These results support the above discussion that the variation of GFIF is satellite-dependent.

**Figure 12:**
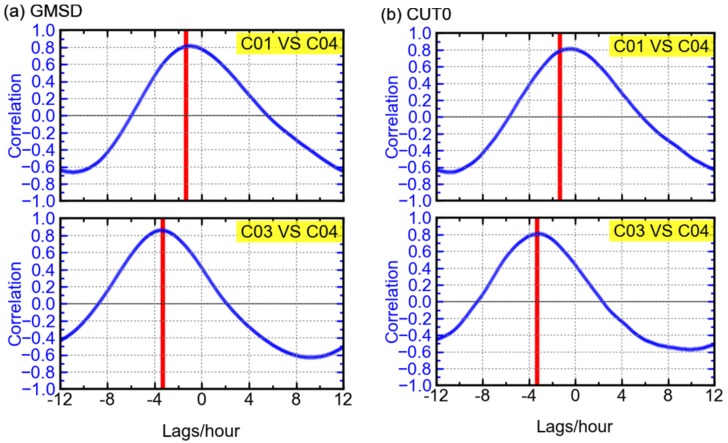
Cross-correlations between the GFIF combination time series (ten days) of different GEO satellites at the stations GMSD (**a**) and CUT0 (**b**), with the red vertical line indicating the local time difference between the two satellites.

Further investigation was conducted to confirm the above. Using observables of ten days, cross-correlation is performed on the GFIF combinations’ time series of different GEO satellites at the stations GMSD and CUT0. As shown in Figure 12, the maximum correlations between the time series of C01 and C04 could reach 0.8. Similar results can be obtained for C03 and C04. The high correlations mean that the patterns of GFIF combinations of the three GEO satellites are almost the same. Furthermore, the peak values of correlation occur when the lag equals to the local time difference between the two satellites. Simply speaking, the local time here indicates the hour angle or, in other words, the relative geometry between the sun and a certain GEO satellite, the latter staying at an almost-fixed longitude. Therefore, the phase shift between GFIF combination time series of each two GEO satellites depends on their longitude difference, suggesting that the GFIF combination of a certain GEO satellite varies according to its relative geometry with the sun. For example, it is likely that the exposure to the sun affects the transmitting hardware at the satellite. Although it is not that explicit or direct, it can provide a possible explanation to the variation in GFIF combinations of BDS GEO satellites.

It is also found that the GFIF time series of C02/C05 show patterns quite different from those of C01/C03/C04 and the correlations between C01/C03/C04 and C02/C05 are low. This may be due to the difference in the satellite manufacturing. The satellites C01/C03/C04 were launched in 2010 and 2011, while the satellites C02/C05 were launched in 2012 [1,23].

## 4. Concluding Remarks

In this paper, GNSS observables from several stations in the Asia-Pacific area were utilized to investigate the CNR, multipath combinations, and geometry-free ionosphere-free combinations of BDS satellites. Their characteristics with time and elevation were analyzed and compared with GPS and Galileo satellites. The CNRs of all except GEO satellites increase significantly with the increase of elevation, and they become stable when the elevation is over 60°. The CNR of B1 observables are the lowest among the three BDS frequencies, while B3 is the highest. The CNR of the GPS L1 observable is remarkably higher than that of L2, while the CNRs of Galileo observables of all three frequencies are at a similar level.

At low elevation angles, the magnitude of code multipath combinations and its dispersion become large. The value of BDS code multipath combination can reach 2.0 m. The code multipath combination of BDS MEO satellites shows “V-shape” variation during a one-day period. Negative correlations are pronouncedly seen between the code multipath combinations and elevations of all BDS non-GEO satellites, validating the elevation-dependent biases. After a single differencing between two stations, the V-shape could be eliminated. This suggests that the V-shape feature in multipath combination is mainly related to the satellites. Proper modelling of the elevation-dependent trend could help correct the measurement and eventually improve the positioning accuracy with BDS.

Diurnal periodicity was found in the GFIF combinations of both BDS GEO and IGSO satellites. The variation range of carrier phase GFIF combinations of GEO satellites is −2.0 to 2.0 cm. The periodicity of carrier phase GFIF combination could be mitigated significantly through a single differencing between two stations. Our analysis indicates that carrier phase GFIF combinations of BDS GEO and IGSO satellites might contain some satellite-related biases. The GFIF time series of C02/C05 show patterns quite different from those of C01/C03/C04. For the BDS GEO satellites C01/C03/C04, the peak values of correlation occur when the lag equals to the local time difference between the two satellites, suggesting that the GFIF combinations’ time series of these satellites might vary according to their relative geometries with the sun.

The above mentioned results are expected to provide useful reference not only for promoting performances of hardware at both satellite and receiver ends, but also for improving algorithms of high-precision positioning with multi-GNSS. On the one hand, more evident experiments are necessary to strictly explain the above phenomena; on the other hand, we are trying to develop and improve the positioning algorithm based on the current knowledge of BDS observables’ characteristics.
